# Bone Marrow Metastasis in Non-hematological Malignancies: Data From a Tertiary Care Hospital in North India

**DOI:** 10.7759/cureus.82757

**Published:** 2025-04-21

**Authors:** Satish Kumar, Kaushal Kumar, Rawi Agrawal, Vijayanand Choudhary

**Affiliations:** 1 Department of Hematology, Indira Gandhi Institute of Medical Sciences, Patna, IND

**Keywords:** bone marrow metastasis, hematological cancer, non-hematological cancer, non-hematological malignancies, retrospective study

## Abstract

Background

It is known that the diagnosis of non-hematological malignancy from bone marrow is uncommon. Also, aspiration from the bone marrow is considered one method that aids in the diagnosis of bone metastases. The study examined bone marrow results and a thorough examination of bone marrow in non-hematological cancers that have metastasized to the bone marrow.

Materials and methods

This was a retrospective study that was conducted at the Department of Hematology, Indira Gandhi Institute of Medical Sciences (IGIMS), Patna, Bihar, India. A total of 200 cases of bone marrow aspiration and 150 cases of bone marrow biopsies with relevant clinical features, hematological, and radiological findings have been enrolled in the study. Ethical clearance has been granted by the Institutional Ethics Committee (IEC) of IGIMS.

Results

It was observed that 62 (31%) patients with non-hematological malignancies suffered from bone marrow metastases. The population with bone marrow metastases varies, as 50 (80.6%) of the patient population comprised adults, while the pediatric population was 12 (19.4%). Anemia, thrombocytopenia, leukopenia, pancytopenia, and nucleated immature blood cells were some common findings of peripheral blood smears associated with non-hematological malignancies.

Conclusion

It concluded the necessity of diagnosing bone marrow metastases in patients with non-hematological malignancies. The study could not find any assured predictive parameter for bone marrow metastases. Conditions like anemia, thrombocytopenia, leukopenia, and others have been revealed by examining peripheral blood smears.

## Introduction

Clinicians have a therapeutic difficulty with metastatic cancer, particularly when treating patients with bone marrow metastases. Nonetheless, it is uncommon for cancer patients to have bone marrow metastases [1, 2]. Mainly, the primary locations of malignancy in those patients who had metastases of bone marrow were the breast, stomach, prostate, and lungs [3, 4].

Bone marrow is often involved in hematological tumors; however, it is rare to diagnose non-hematological cancers from the bone marrow alone. Patients with metastases of the bone marrow experience anemia and cytopenia as their initial signs and symptoms frequently [5]. To determine the stage, prognosis, treatment plan, and chemotherapy responsiveness of the malignancy, it is essential to look for metastatic deposits in the bone marrow [6].

Both the advanced stage of the disease and a bad prognosis are indicated when a non-hematologic malignancy spreads to the bone marrow [7]. Aspiration testing of the bone marrow helps detect bone metastases early. Still, bone marrow metastasis is difficult to find in bone marrow because bone marrow aspiration procedures are not routinely performed in the examination of cancer patients [8, 9].

Since all potential treatment plans have been exhausted or because they are too frail to undergo any anticancer therapy, many patients with bone marrow metastases only receive supportive care [10]. Bone marrow metastasis is now much more likely to be detected thanks to the recent development of sensitive and new diagnostic techniques like reverse transcriptase polymerase chain reaction and flow cytometry, which are used to identify spread tumor cells [11, 12]. The study was performed to assess the hematological results and thorough bone marrow investigation in cases of nonhematological cancers that have spread to the bone marrow.

## Materials and methods

Study design

This was a retrospective study. Data from four years and eight months, i.e., from May 2019 to January 2024, have been collected for the study. The study was conducted at the Department of Hematology, Indira Gandhi Institute of Medical Sciences (IGIMS), Patna, Bihar, India.

Study population

A total of 200 patients with non-hematological malignancy were enrolled in the study. At the time of initial presentation, a bone marrow examination was conducted on patients with non-hematological malignancies. The patients excluded were individuals receiving chemotherapy or having hematopoietic or lymphoid malignancy. Also, the cases with incomplete records were excluded from the final study.

Study procedure

Immunohistochemistry, flow cytometry, defined morphology, and the pattern of metastatic tumor cells with radiological and clinical data all supported the diagnosis of bone marrow metastasis in non-hematological malignancy. Some of the relevant images of immunohistochemistry, defined morphology, and pattern of metastatic tumor cells have been described below.

**Figure 1:**
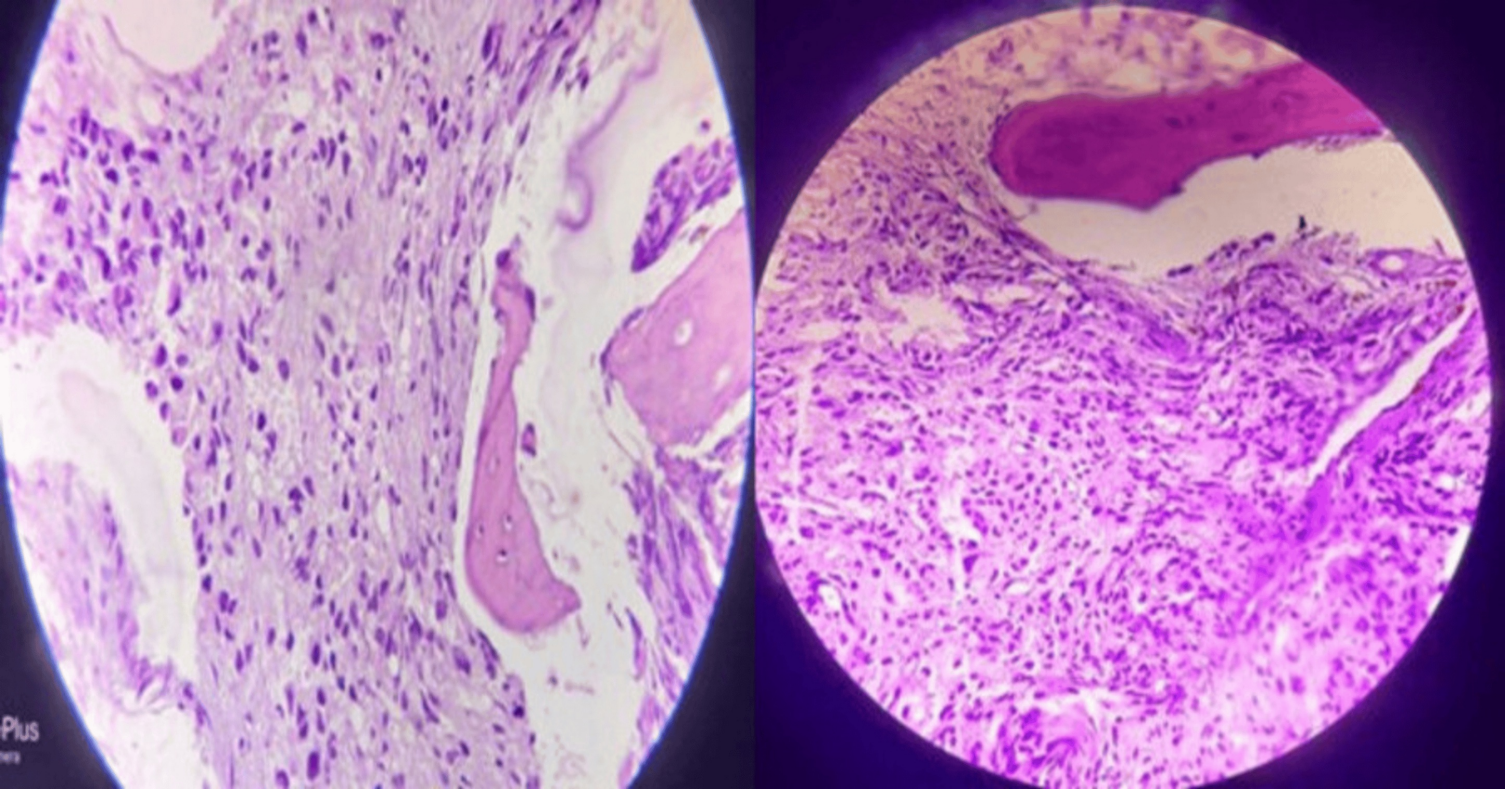
Hematoxylin and eosin-stained bone marrow biopsy showing adenocarcinoma of the prostate (×40) (left) and adenocarcinoma of the lung (×40) (right).

**Figure 2:**
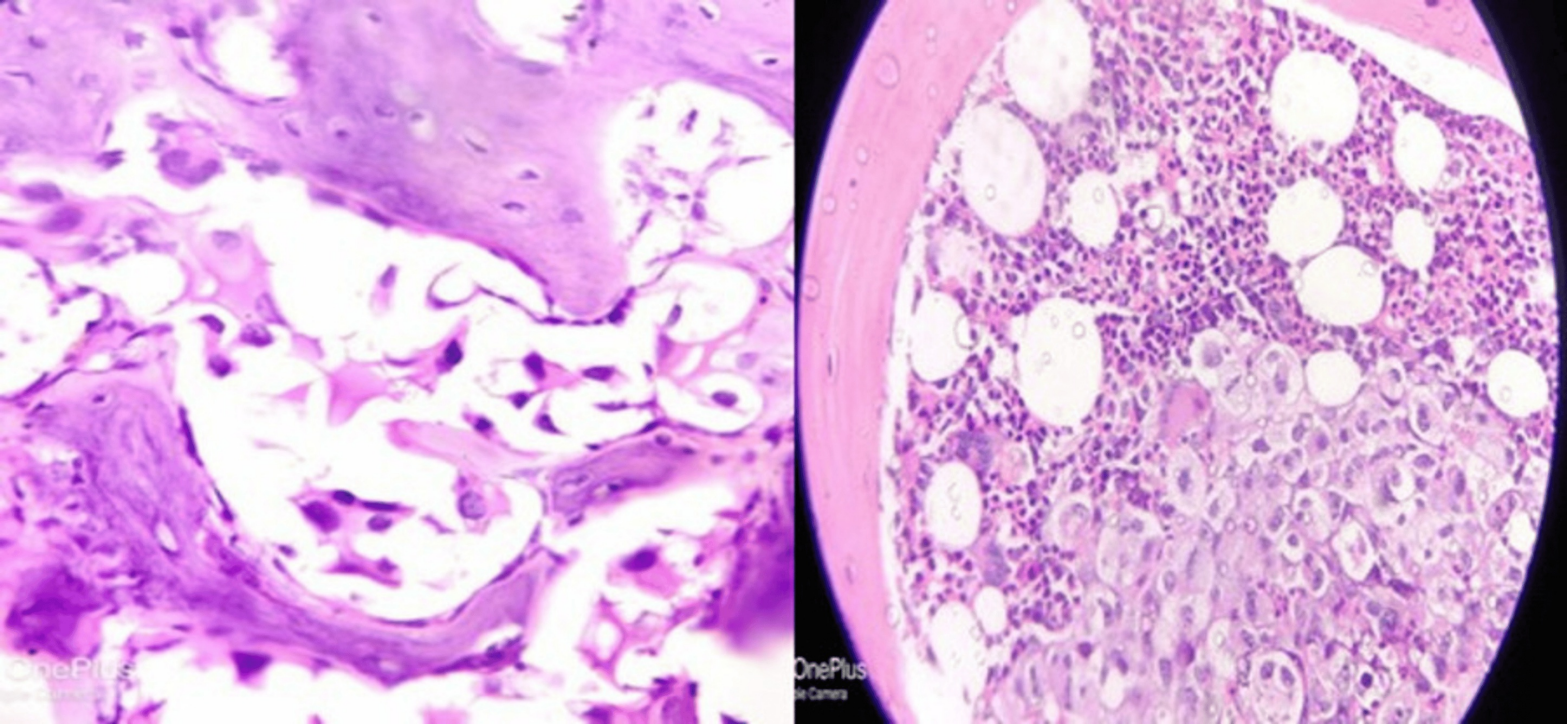
Hematoxylin and eosin-stained bone marrow biopsy showing poorly differentiated carcinoma (×40) (left) and metastatic deposits of malignant epithelial tumor of the gastrointestinal tract (GIT) (×40) (right).

**Figure 3:**
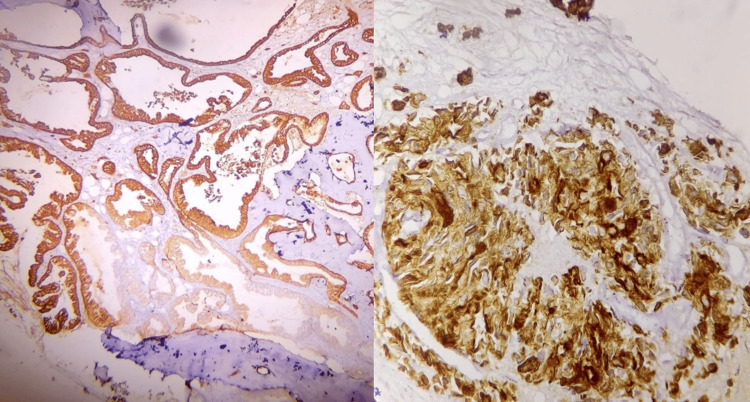
Immunohistochemistry of bone marrow biopsy showing CK7+ in metastatic deposit of adenocarcinoma of the gastrointestinal tract (GIT) (x40) (left) and PanCK+ in metastatic deposit of adenocarcinoma of lung (x40) (right).

Figure 1 shows a hematoxylin and eosin-stained bone marrow biopsy showing adenocarcinoma of the prostate (left) and lung (right), respectively. Figure 2 shows poorly differentiated carcinoma (×40) (left) and metastatic deposits of malignant epithelial tumor of the gastrointestinal tract (GIT) (×40) (right). Lastly, Figure 3 shows immunohistochemistry of bone marrow biopsy showing CK7+ in metastatic deposit of adenocarcinoma of the GIT (x40) (left) and PanCK+ in metastatic deposit of adenocarcinoma of the lung (x40) (right).

Statistical analysis

Microsoft Excel (Microsoft Corp., Redmond, WA) was initially used to enter the data. Data were then analyzed using IBM SPSS Statistics software version 21.0 (IBM Corp., Armonk, NY). Conventional descriptive statistics were performed in the present study. An independent t-test was used to obtain the t-scores and p-value. Data have been presented as either n or n (%). The p-value was considered significant at <0.05.

Ethical approval

Ethical clearance has been granted by the Institutional Ethics Committee of IGIMS, dated 20 March 2024, under letter number 1368/IEC/IGIMS/2024.

## Results

A total of 4,442 bone marrow aspirations and 3,268 bone marrow biopsies were done during a period of four years and eight months from May 2019 to January 2024. Out of which 200 were done to rule out bone marrow metastasis in cases of non-hematological malignancies. Out of 200 patients with non-hematological malignancies, it was found that 62 (31%) patients had bone marrow metastases. Smears of aspiration of bone marrow, smears of peripheral blood, biopsies of bone marrow, and other records were examined in greater detail for instances involving bone marrow involvement. Out of 62 patients, 40 (64.5%) were male and 22 (35.5%) were female. Fifty (80.6%) were adult patients, and 12 (19.4%) were in the pediatric age group in bone marrow metastases cases. The median age for adult patients was 54.7 years, and for pediatric patients, it was 10 years. Table 1 represents the frequencies of bone marrow metastasis among the study patients.

**Table 1:** Data on metastases from bone marrow in the study population Data are presented as n (%); p-value was considered significant at <0.05; an independent t-test was used to obtain the t-score and p-value.

Groups	Adult population	Pediatric population	t-score	p-value
Non-hematological malignancies (n=200)	161 (80.5%)	39 (19.5%)	-12.2	<0.0001
Bone marrow metastases (n=62)	50 (80.6%)	12 (19. 4%)	-6.8	<0.0001

**Figure 4:**
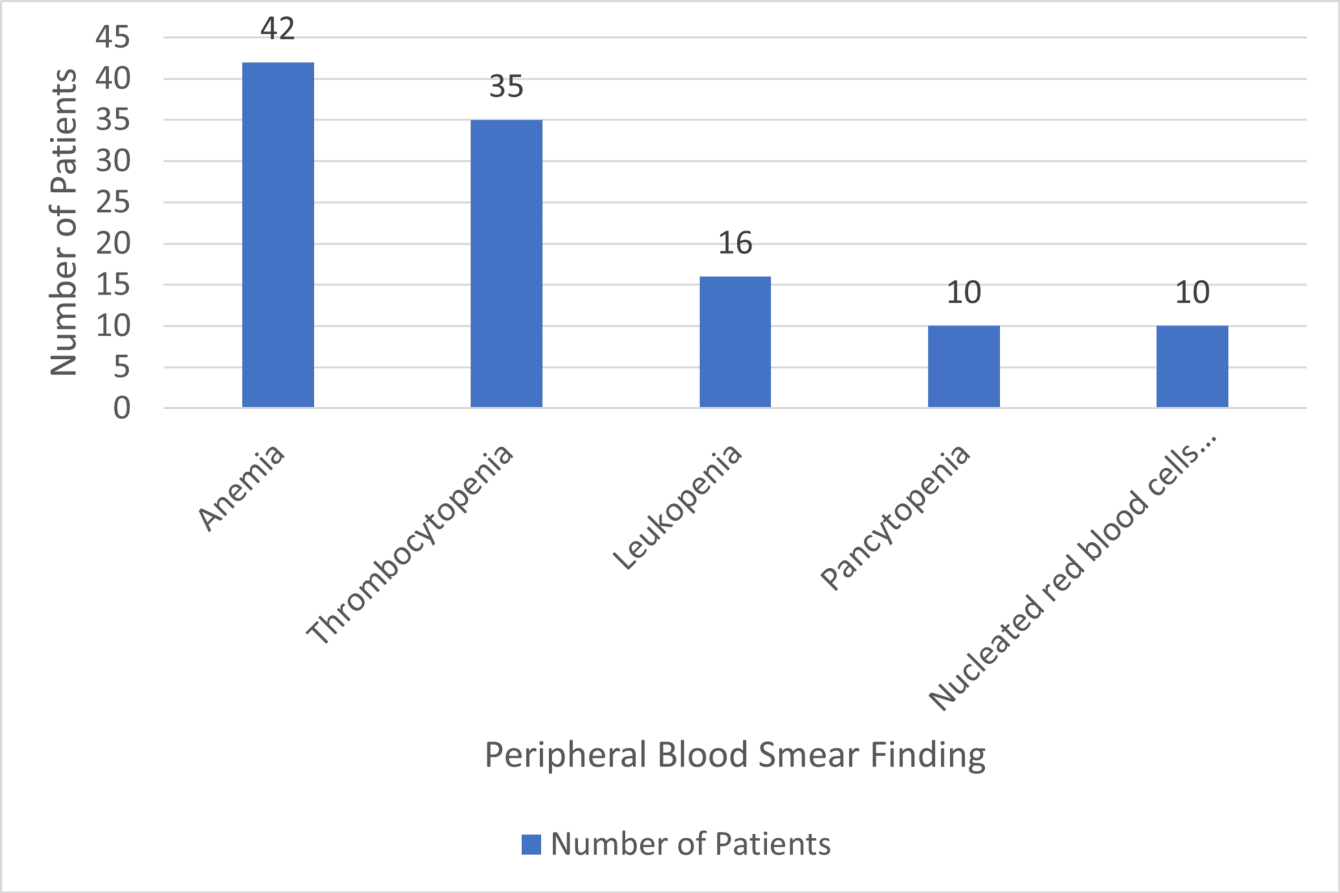
Peripheral blood smear findings among participants

Figure 4 represents clinical hematomorphological findings in non-hematological malignancies with bone marrow metastases. It has been found that anemia is the most common hematological finding associated with bone marrow metastases, which is present in approximately 42 (67.7%) of patients. The frequency of leukopenia and pancytopenia was lower in terms of cases. However, the presence of immature or nucleated red blood cells constituted another important characteristic of non-hematological cancers.

Table 2 presents the incidence of adult cases of bone marrow metastases. The majority of patients had lung cancer, which was followed by non-hematological cancers of the GIT, prostate, breast, ovary, and bladder. In adults, gastrointestinal carcinoma was a prevalent cancer with bone marrow metastases (14/29 or 48.27%).

**Table 2:** Bone marrow metastases in adult patients Data are presented as n/n (%).

Primary tumor	Number of cases with bone metastases/total number of cases present (%)
Lung cancer	16/38 (42.10%)
Breast cancer	06/30 (20.00%)
Prostate cancer	10/38 (26.31%)
Gastrointestinal cancer	14/29 (48.27%)
Ovarian cancer	02/06 (33.3%)
Bladder cancer	02/20 (10.00%)

Table 3 presents data on bone marrow metastases in pediatric patients. Generally, neuroblastoma and retinoblastoma are commonly found as malignancies. Retinoblastoma was found to be significant with a p-value of 0.01 between patients with metastases of bone and patients without metastases of bone.

**Table 3:** Bone marrow metastases in pediatric patients Data are presented as n (%); p-value was considered significant at <0.05; an independent t-test was used to obtain the t-score and p-value.

Primary tumor	Number of cases with bone metastases	Number of cases without bone metastases	t-score	P-value
Neuroblastoma (n=9)	4 (44.4%)	5 (55.6%)	0.47	0.32
Retinoblastoma (n=30)	8 (26.7%)	22 (73.3%)	3.6	0.01

## Discussion

In the present study, 31% of patients with non-hematological malignancies had bone marrow metastases. Although it is very uncommon to have metastases of bone marrow along with non-hematological malignancies. The involvement of bone marrow is commonly seen in hematopoietic disorders. Bone marrow metastasis by nonhematological malignancy is rare. An advanced stage with a poor prognosis is indicated by the involvement of non-hematologic solid tumors. For staging, prognosis, and treatment, it is critical to identify bone marrow involvement by solid malignancies [13]. When there is no known cause, bone marrow involvement might occasionally be the initial sign of the illness. In children, retinoblastoma, neuroblastoma, Ewing's sarcoma, rhabdomyosarcoma, and medulloblastoma account for the majority of encountered metastases. Also, as per studies, bone marrow examination seems to be a more useful and economical method [14]. In adults, lymphoma, carcinoma, prostate, breast, lung, GIT, and female genital tract are among the tumors that frequently spread to the bone marrow [15]. In known cases of cancer, trephine biopsy and bone marrow aspiration are performed. Additionally, it is performed when there are radiographic signs that point to bone marrow involvement, unexplained cytopenias, and abnormal peripheral blood results.

Numerous research studies on bone marrow invasion by non-hematological malignancies have been conducted in the past. The most frequent hematological abnormalities found were leucoerythroblastic blood image, thrombocytopenia, and anemia [7]. Similar results were obtained from the research by Kopp et al., which visibly exhibited the metastases of bone marrow [16].

A study done by Singh et al. shows that tumor cells in bone marrow cause marked stromal fibrosis and yield a dry tap on aspiration. Therefore, it is best to perform aspiration and biopsy of the bone marrow at the same time. It also found that sometimes trephine biopsy of the bone marrow failed to detect metastasis of the bone marrow [14].

Our study showed anemia as the most prevalent hematological finding in non-hematological malignancies. According to a study by Sar et al., anemia is the most prevalent kind found in peripheral blood smears. Their findings demonstrated that leukopenia occurred in 50% of instances and thrombocytopenia occurred in 83.3% of patients with metastatic bone marrow involvement [17]. All of the patients with metastases of bone marrow experienced anemia and thrombocytopenia, according to the study conducted by Ozkalemkas et al. [2]. Furthermore, both anemia and thrombocytopenia were found among 71.4% and 45.1% of Mehdi and Bhatt's patients with bone marrow metastases, respectively [3].

Our study included non-hematological malignancies in cancers like lung, breast, prostate, gastrointestinal, ovarian, and bladder cancer in adults, while neuroblastoma and retinoblastoma were in children. Other studies have found that the most common initial non-hematological malignancies in persons with bone marrow metastases are those of the breast, lung, stomach, and prostate. Neuroblastoma, rhabdomyosarcoma, and retinoblastoma are the three most prevalent cancers in children [6, 18].

The common primary tumor in non-hematological malignancies in our study was lung cancer, followed by gastrointestinal, prostate, breast, bladder, and ovarian cancer in adults. While an association of non-hematological malignancies with bone marrow metastases was more frequently seen in gastrointestinal (48.27%), lung (42.10%), ovary (33.3%), prostate (26.3%), breast (20.0%), and bladder (10.0%). According to a related study by Mehdi et al., adenocarcinoma of the prostate (36%) was the most frequent cause of bone marrow metastasis, followed by adenocarcinoma of the stomach (25%) and breast cancer (22.2%). [3].

On the other hand, in the pediatric population, children with non-hematological malignancies were more affected by neuroblastoma and retinoblastoma. While the association of non-hematological malignancies with bone marrow metastases was mainly present in children with neuroblastoma (44.4%), followed by retinoblastoma (26.6%).

Limitations

The limitations of the study are that the study was retrospective; additionally, as the cases of bone metastases were less prevalent, the duration of the study was shorter. If data for a longer duration had been collected, they could have reported significant results.

## Conclusions

The study concludes that it is necessary to diagnose metastases of the bone marrow in non-hematological malignancies, which can help in tumor staging, therapy selection, and risk of disease prognosis. The study showed statistically significant differences in the adult population and pediatric population in both the respective cases of non-hematological malignancies and bone marrow metastases. As a blood disorder, anemia is found to be commonly associated with bone marrow metastases. Peripheral blood smear evaluation showed clinical bone marrow examination in terms of conditions like anemia, thrombocytopenia, leukopenia, pancytopenia, and non-nucleated immature blood cells. Lung cancer was observed to be a prominent primary tumor in adult cases with bone marrow metastases.
